# Management of take-all disease caused by *Gaeumannomyces graminis* var. *tritici* in wheat through *Bacillus subtilis* strains

**DOI:** 10.3389/fmicb.2023.1118176

**Published:** 2023-02-01

**Authors:** Gangyi Zhao, Tianjie Sun, Zina Zhang, Jingjing Zhang, Yinbo Bian, Chunyan Hou, Dongdong Zhang, Shengfang Han, Dongmei Wang

**Affiliations:** ^1^State Key Laboratory of North China Crop Improvement and Regulation, Baoding, China; ^2^Key Laboratory of Hebei Province for Plant Physiology and Molecular Pathology, Baoding, China; ^3^College of Life Sciences, Hebei Agricultural University, Baoding, China

**Keywords:** take-all disease, *Bacillus subtilis*, biological control, combination of strains, antimicrobial protein

## Abstract

Wheat (*Triticum aestivum*) is the second largest grain crop worldwide, and one of the three major grain crops produced in China. Take-all disease, caused by *Gaeumannomyces graminis* var. *tritici* (*Ggt*) infection, is a widespread and devastating soil-borne disease that harms wheat production. At present, the prevention and control of wheat take-all depend largely on the application of chemical pesticides. Chemical pesticides, however, not only lead to increased drug resistance of pathogens but also leave significant residues in the soil, causing serious environmental pollution. In this study, we investigated the application of *Bacillus subtilis* to achieve take-all disease control in wheat while reducing pesticide application. Antagonistic bacteria were screened by plate test, species identification of strains was performed by Gram staining and sequencing of 16s rDNA, secondary metabolite activity of strains was detected by clear circle method, strain compatibility and effect of compounding on *Ggt* were detected by plate, and the application prospects of specific strains were analyzed by greenhouse and field experiments. We found that five *B. subtilis* strains, JY122, JY214, ZY133, NW03, Z-14, had significant antagonistic effects against *Ggt*, and could secrete antimicrobial proteins including amylase, protease, and cellulase. Furthermore, Z-14 and JY214 cultures have also been shown to change the morphology of *Ggt* mycelium. These results also showed that Z-14, JY214, and their combination can control take-all disease in wheat at a reduced level of pesticide use. In summary, we screened two *Bacillus* spp. strains, Z-14 and JY214, that could act as antagonists that contribute to the biological control of wheat take-all disease. These findings provide resources and ideas for controlling crop diseases in an environmentally friendly manner.

## Introduction

Wheat is one of the main food crops produced in China, and the most important crops worldwide (Shewry and Hey, 2015). Wheat take-all is a serious global wheat rhizosphere disease caused by the *Gaeumannomyces graminis* var. *tritici* (*Ggt*) infection. *Ggt* infests wheat throughout the growing period, with increased yellow leaves on affected plants at the seedling stage, delayed return of diseased seedlings in winter wheat, few tillers, weak growth and dwarfing, and the most pronounced symptom expression at maturity. The degree of soil moisture also affects the incidence of allograft. This disease can also produce disorders in dry areas, but the disease is aggravated under moist soil conditions. Severe cases of take-all disease have caused continuous wheat blight, resulting in loss of yield and quality (Freeman and Ward, 2004; Palma-Guerrero et al., 2021). Because of the extreme difficulty in preventing and controlling wheat take-all disease, it has been called the “cancer of wheat.” Even at the moment, due to the lack of effective disease-resistant cultivars, the control of wheat take-all relies largely on the prolonged use of pesticides that lead to environmental pollution and increased resistance of the pathogen. Considering the unsatisfactory effectiveness of existing biocontrol formulations against *Ggt*, combining the application of pesticides in reduced dosage with the use of biocontrol formulations with antagonistic effects is becoming a promising strategy (Yu, 2017). Microbial biocontrol agents are deemed to be environmentally friendly, and can replace chemical agents to a certain extent in disease control (Zhang et al., 2021a). Commonly used biocontrol microorganisms currently include bacterial *Pseudomonas* spp. and *Bacillus* spp., and fungal *Trichoderma, Simplicillium*, and *Nigrospora* (Kaur et al., 2006; Yang et al., 2011, 2018; Jiang and Ding, 2016). A large number of studies have been conducted in recent years on the application of biological control agents for the management of plant pathogens. Carola et al. showed that the combination of *Pseudomonas protegens* strains with the chemical agent fluoroquinazole has significant effect to control take-all disease (Vera Palma et al., 2019). Application of the biocontrol strain SC11 combined with strains 153 and Fo47 showed a high control efficiency (89.47%) on *Fusarium wilt* infection in watermelon (Lan et al., 2015). The results of Sun et al. showed that the inhibition efficiency of *Bacillus amyloliticus* SW-34 suspension on gray mold was 72.29% in ginseng, which was significantly higher than that of fungicide treatment (Sun et al., 2019). It has been found that *Bacillus* could suppress pathogenic microorganisms in multiple ways, such as by secreting secondary metabolites that damage the cell membranes of pathogenic organisms, by blocking energy metabolic pathways of pathogen cells, by competing with pathogens for ecological sites, by hindering pathogen infestation, and also by producing growth-promoting substances to accelerate plant growth or by inducing systemic resistance in plants (Tanaka et al., 2017; Fira et al., 2018; Lin et al., 2018; Yang, 2019). For instance, it was reported by Myo et al. (2019) that *Bacillus velezensis* strain NKG-2 inhibits the growth of various pathogenic fungi including *Fusarium oxysporum* through producing volatile substances. *Bacillus cereus* strain AR156 colonized the rhizosphere and occupied the loci, and effectively inhibited the infection of *Ralstonia solanacearum* (Wang et al., 2014a). *Bacillus amyloliquefaciens* strain FS6 colonized in ginseng and exerted its role in enhancing disease resistance by inducing the activity of defense-related enzymes (Wang et al., 2019). However, there are only limited studies on the role of *Bacillus subtilis* in controlling take-all disease (Zhang et al., 2021b). In this study, we screened for microorganism with inhibitory effect to the growth of *Ggt*, and found that four *B. subtilis* strains and the *B. amyloliquefaciens* strain Z-14 could act as antagonists of *Ggt* growth. Among which, two strains could promote the growth of wheat seedlings. Our findings could be used in the biocontrol of take-all diseases in wheat, with the aim of reducing pesticide use and promoting wheat growth.

## Materials and methods

### Cultivation conditions for bacterial and fungal strains

Bacterial strains JY122, JY214, ZY133, NW03, NO36, and JY215 were isolated for both cellulose and lignin degradation availability, from soil near wheat roots in different rural areas in Tangshan, Cangzhou, Daming, Zhengding, and Xingtang in Hebei province, China (Xiong et al., 2020; Zhang et al., 2021c). Bacterial strains were cultivated in NB medium (10 g/L peptone, 5 g/L beef extract, and 10 g/L sodium chloride, pH 7.3) at 37°C, according to a published protocol (Tao et al., 2021), and *Ggt* was cultivated in PDA medium at a constant temperature of 26°C.

### Screening of antagonists and observation of *Ggt* mycelium

The procedure for the analysis of the inhibitory effect of different *Bacillus* spp. on *Ggt* growth was carried out according to previous reports (Zhang et al., 2014a; Wang et al., 2021). The identical amount of *Ggt* mycelium were inoculated in the central area of the dishes with PDA medium and the bacterial cultures (which were pre-cultivated till OD_600_ = 1.0) were inoculated on the medium 2.5 cm apart from the *Ggt* inoculum and subsequently incubated at 26°C. The experiment was repeated three times, with each containing three technical replicates. Upon extension of mycelium to the edge of the dishes in the control group which was inoculated with *Ggt* alone, the radius of *Ggt* mycelium on the dishes inoculated with different *Bacillus* spp. were measured and the inhibition rate was calculated. Inhibition rate = (the radius of mycelial expansion area of the control group – the radius of mycelial expansion area of the test group) / the radius of mycelial expansion area of the control group × 100%.

### Bacterial species identification

Gram staining was carried out according to the method mentioned in a previously reported protocol (Tian et al., 2008). The regions of 16S rDNA of the strains to be identified were amplified with general primers 27F: 5′-AGAGTTTGATCCTGGCTCAG-3′ and 1492R: 5′-TCGCAATATGATCACGGCTA-3′. Bacterial strains cultivated in NB medium to OD_600_ = 1.0 were used directly as templates for PCR analysis using 2 × rapid taq master mix (Vazyme, China). The thermocycling conditions were 95°C for 5 min (initial denaturation) followed by 35 cycles of 95°C 1 min (denaturation); 55°C 30 s (annealing); and 72°C 1 min 30 s (extension), 72°C 10 min (final extension). PCR products were purified and sequenced using primers that were used to amplify them. The sequencing results of the PCR products were analyzed by using BLAST program against the nucleotide collection (nr) database from NCBI, and the sequence homology was compared with that of the model strains. The phylogenetic tree was constructed by using MEGA7.0 software (Kumar et al., 2016). The similarity between nucleic was inferred by using the Maximum Likelihood method and JTT matrix-based model. The phylogenetic tree was obtained automatically by applying Neighbor-Join and BioNJ algorithms to a matrix of pairwise distances estimated using a JTT model.

### Detection of protease, amylase, and cellulase activities in bacterial secondary metabolites

Bacterial cultures were inoculated into a seed medium (10 g/L peptone, 10 g/L glucose, 2 g/L sodium phosphate monobasic dihydrate, and magnesium sulfate heptahydrate) at a ratio of 1:100 (v:v), cultivated at 37°C for 48 h with shaking, centrifuged, and filtered with a 0.22 μm filter. The filtrate was dropped into the corresponding medium for enzymatic activity detection, and incubated at 37°C for 36–48 h.

Protease activity was detected on skim milk agar plates (Yang et al., 2008). Amylase activity was detected by starch medium, and compound iodine solution was used for color development (Liu et al., 2010; Wang et al., 2014b). Cellulase activity was detected on carboxymethyl cellulose agar plates (Saritha et al., 2012; Tokpah et al., 2016).

### Detection of compatibility and combination ratio between bacterial strains

The bacterial cultures were evenly coated on plates with NA medium, and the culture of the bacteria to be tested were dropped on it. The medium was incubated at 37°C for 24 h. The appearance of transparent ring around the bacterial inoculation site indicates the incompatibility of the two tested bacteria.

We employed two different mixing approaches to test the inhibitory effect of the combination of two different *Bacillus* spp. strains on *Ggt*, namely mix after-cultivation and co-cultivation. The mix after-cultivation refers to the mixing of two *Bacillus* spp. strains that were pre-cultured in NB medium until OD_600_ = 1.0, while co-cultivation refers to inoculating a mixture of two *Bacillus* spp. strains into 100 times the volume of NB medium.

### Assessment of the control effect of bacterial strains against *Ggt* infection

The preventive and control effects of the *Bacillus* spp. strains against *Ggt* infection were tested in the greenhouse and in the field. The pesticide used in this study was Koras (27% Thiamethoxam-Cymoxanil-Phenoxymethoxazole, produced by Syngenta, Beijing). In the greenhouse experiments, 50 g of wheat seeds was treated with 1.8% pesticide diluted in dH_2_O, while in the field experiments, 150 g of wheat seeds was treated with 5% pesticide diluted in dH_2_O. The greenhouse test consists of ten treatments: treatment 1: Nutrient soil + sterile water, treatment 2: Nutrient soil + *Ggt* + sterile water, treatment 3: Nutrient soil + *Ggt* + 1.8% pesticide + sterile water, treatment 4: Nutrient soil + *Ggt* + Z-14 culture solution, treatment 5: Nutrient soil + *Ggt* + JY214 culture solution, treatment 6: Nutrient soil + *Ggt* + mixture of Z-14/JY214 culture solution, treatment 7: Nutrient soil + *Ggt* + 0.9% pesticide + sterile water, treatment 8: Nutrient soil + *Ggt* + 0.9% pesticide + Z-14 culture solution, treatment 9: Nutrient soil + *Ggt* + 0.9% pesticide + JY214 culture solution, treatment 10: Nutrient soil + *Ggt* + 0.9% pesticide + mixture of Z-14/JY214 culture solution. Plant pots with a diameter of 12.9 cm and a height of 12.4 cm were used in this study. Eight wheat seeds were sown in each pot, and the test was done in three replicates. The pathogen, *Ggt*, was inoculated by placing pre-cultivated hyphal sheets (7 mm in diameter) on the soil, one wheat seed was placed on each sheet, and irrigated with 30 mL of antagonistic solution containing the bacteria to be tested. Two weeks after germination, the seedlings were irrigated again with antagonistic solution. The stem width, plant height, and root length were measured at about 40 days after germination, and then the average values were calculated. The disease index was calculated depending on the severity of *Ggt* infestation in wheat seedlings. The disease index was set in five grades according to the proportion of wheat seedlings that were infected with *Ggt*, according to a previous report (Qiao et al., 2005). Grade 0 = 0%, grade 1 = 1–10%, grade 2 = 11–30%, grade 3 = 31–60%, and grade 4 = 61–100%.

The disease index and disease reduction were calculated according to a report by Chen et al. (2021).

Disease index = [∑ (number of infected plants at all levels × representative value)/total number of investigated plants × representative value of the heaviest disease level] × 100%.

Disease reduction rate = [(control group disease index – treatment group disease index)/control group disease index] × 100%.

The field test was conducted in the experimental field of the meteorological station in Hebei Agricultural University (West campus, Baoding, China). The land used for the experiment had been planted with winter wheat and summer corn for several years. A randomized group design was used for each experiment treatment. All plots were separated and protected with protection rows, compound fertilizer and urea were applied before sowing. The wheat was sowed in October, 2021, and harvested in June, 2022, and the agronomic parameters were recorded at the stage of maturity.

The field test was set up with eight treatments: treatment 1: Blank control, treatment 2: *Ggt*, treatment 3: *Ggt* + 5% pesticide, treatment 4: *Ggt* + 5% pesticide +Z-14 culture solution, treatment 5: *Ggt* + 5% pesticide + JY214 culture solution, treatment 6: *Ggt* + 5% pesticide + mixture of Z-14/JY214 culture solution, treatment 7: *Ggt* + 2.5% pesticide + Z-14 culture solution, treatment 8: *Ggt* + 2.5% pesticide + mixture of Z-14/JY214 culture solution. The pathogen was inoculated by grinding the *Ggt*-infested wheat into powder and spreading it evenly on the soil surface of the field. The bacteria were inoculated by centrifuging the culture solution and mixing the precipitate with wheat seeds, and the pesticide was applied similarly in a mixed manner, according to a previous report (Wang et al., 2021).

## Results

### *Ggt* was inhibited by certain *Bacillus* spp. strains

**Figure 1:**
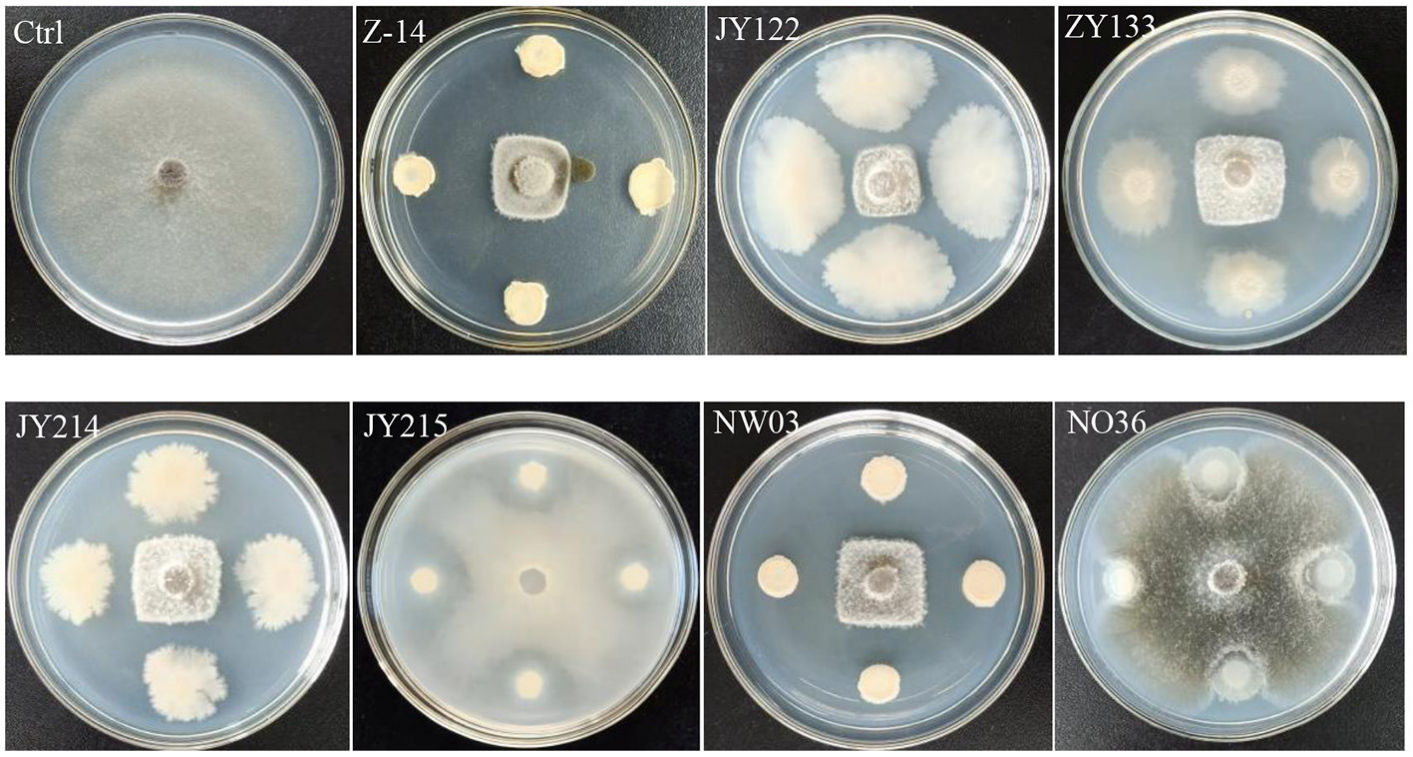
Antagonistic effect of the selected bacteria strains against *Ggt*. The *Ggt* were inoculated in the center of PDA plate, and the strains to be tested were inoculated around the *Ggt*-inoculation sites.

Antagonistic effects of the screened strains on the pathogen of wheat take-all disease, *Ggt*, were detected by plate antagonistic test. The results showed that strains Z-14, JY122, ZY133, JY214, and NW03 had significant antagonistic effects on the growth of *Ggt*, while JY215 and NO36 showed only slight inhibition on the growth of *Ggt* (Figure 1). The inhibition rate of Z-14 to *Ggt* was as high as 77.58%, and the inhibition rate of JY214 was 73.02% (Table 1). Five strains with good antagonistic effects against wheat take-all were obtained.

**Table 1:** Inhibition rates of seven bacterial strains against *Ggt*.

**Inoculum**	**Radius of the bacterial colony (cm)**	**Inhibition rate (%)**
*Bacillus subtilis* Z-14	0.94 ± 0.01a	77.58 ± 0.34c
*Bacillus subtilis* JY122	0.96 ± 0.06a	77.18 ± 1.50c
*Bacillus subtilis* ZY133	1.17 ± 0.04b	72.22 ± 0.91b
*Bacillus subtilis* JY214	1.13 ± 0.01b	73.02 ± 0.34b
*Bacillus subtilis* JY215	2.39 ± 0.06a	43.06 ± 1.37a
*Bacillus subtilis* NW03	0.98 ± 0.04c	76.79 ± 1.03c
*Bacillus subtilis* NO36	2.33 ± 0.04c	44.64 ± 1.03a
CK	-	-

Values in the table are mean ± standard deviation. Different letters indicate the significant differences (*P* < 0.05 by one-way ANOVA and Duncan's multiple comparison).

### Antagonistic bacterial strains JY214, JY122, ZY133, and NW03 were identified to be *Bacillus subtilis*

**Figure 2:**
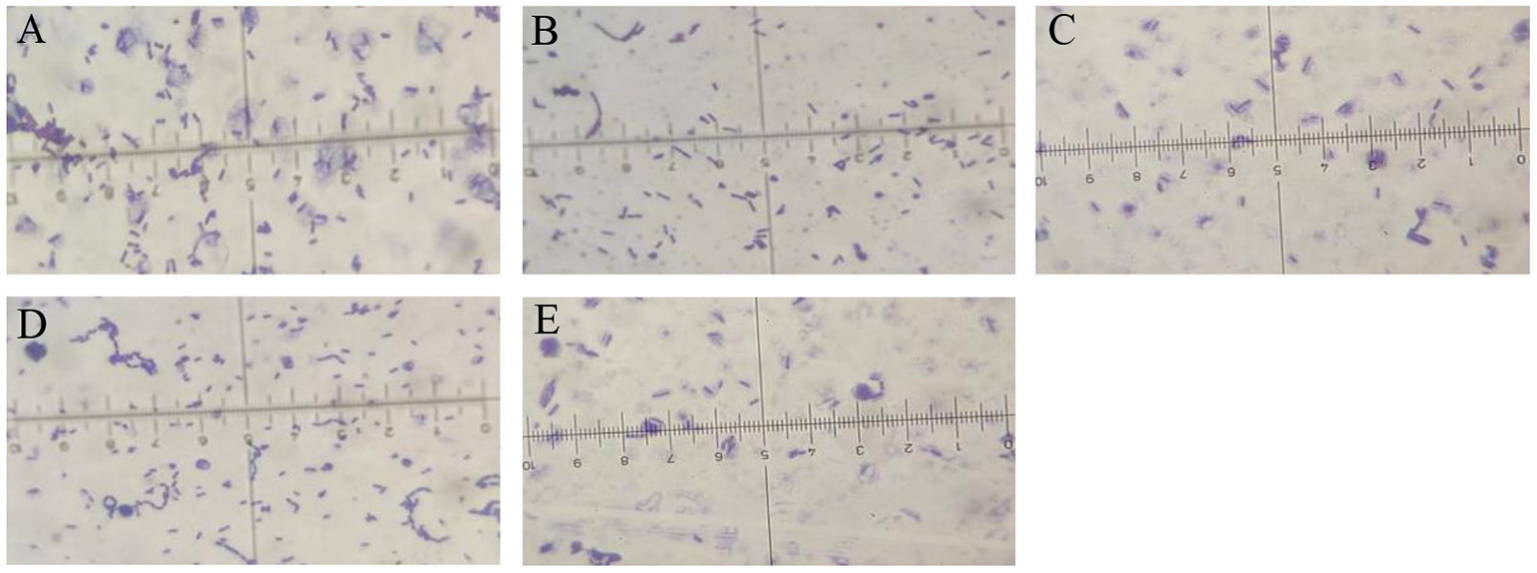
Microscopic images of the selected bacterial strains. Gram stain was used to enhance the visibility of the bacteria. **(A)** Z-14, **(B)** JY122, **(C)** ZY133, **(D)** JY214, and **(E)** NW03.

**Figure 3:**
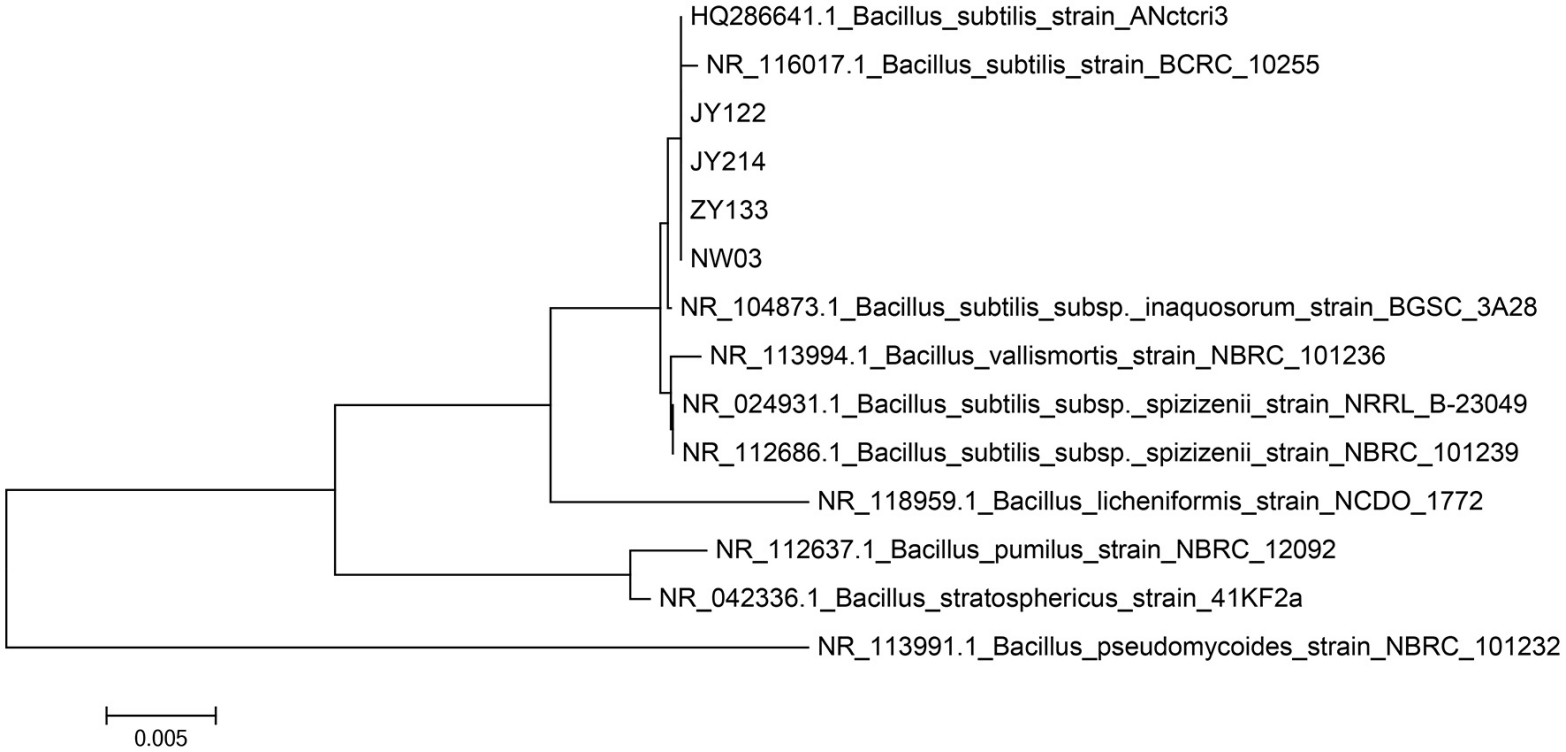
Phylogenetic analysis of 16s rDNA sequencing results of antagonistic strains. Nucleic sequences from several stains were used for phylogenetic analysis using the maximum likelihood method and JTT matrix-based model. The scale bar indicates the number of nucleic acid substitutions per site.

Gram staining and 16s rDNA fragment sequencing were performed on the broth to identify the species of the strains specifically. The strains were identified as Gram-positive as they showed a purplish color after Gram staining (Figure 2) and a short rod-shaped body was observed under the microscope. The 16s rDNA of the antagonistic bacteria was amplified, and the sequencing results of the PCR products were compared in the NCBI database for Blast comparison. The results of phylogenetic analysis showed that strains JY214, JY122, ZY133, and NW03 were clustered into a branch, which had a high similarity with *B. subtilis* strains (Figure 3).

### Activities of protease, amylase and cellulase in secondary metabolites of the antagonistic strains

**Figure 4:**
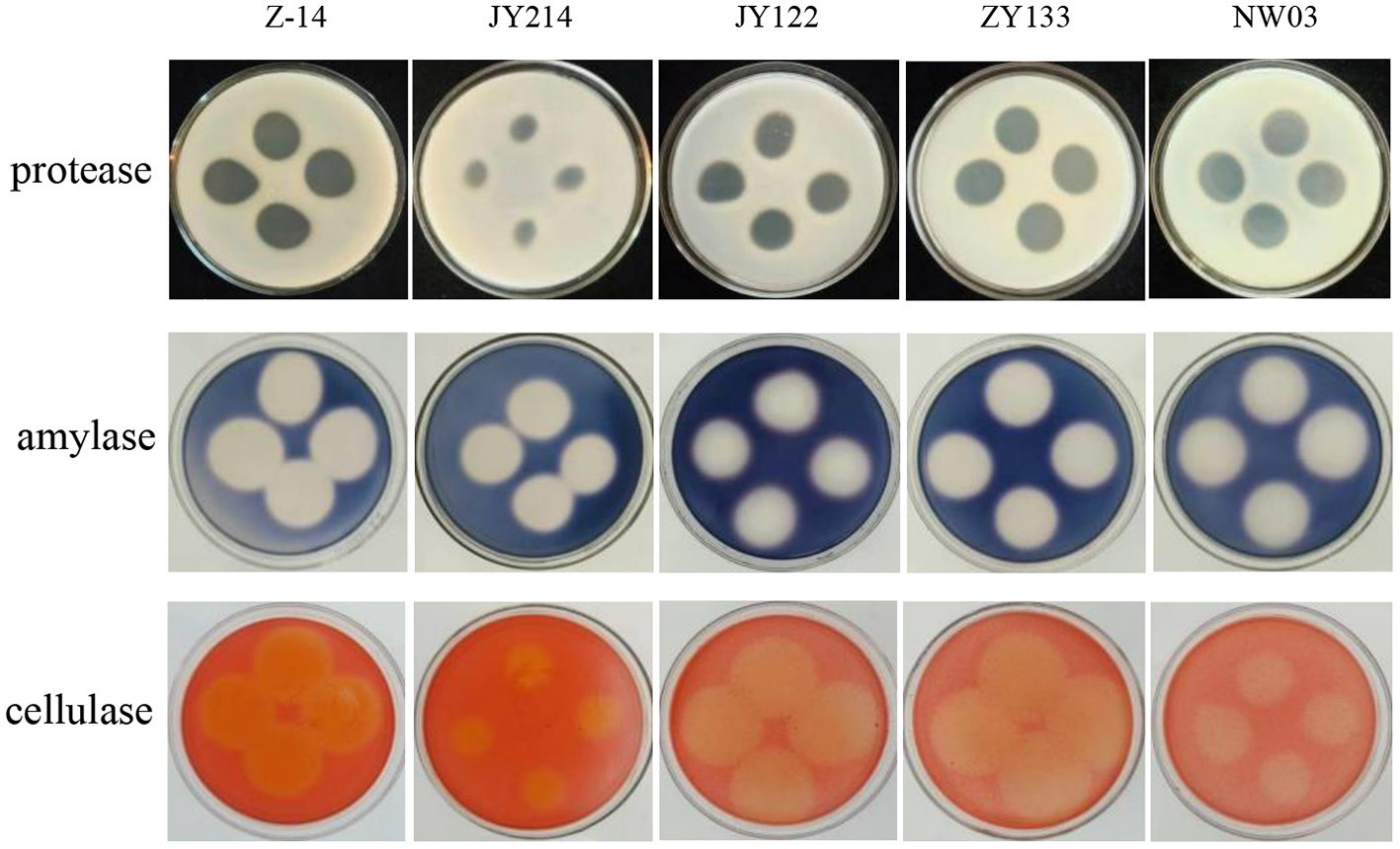
Secondary metabolite detection. Medium containing milk, starch, and sodium carboxymethyl cellulose were used to detect protease, amylase, and cellulase activities, respectively. Secondary metabolites were dropped and cultured at 37°C for 48 h.

The activity of protease, amylase, and cellulase in the metabolites of antagonistic strains were detected. The results showed that clear rings of different sizes were present in the test plates, indicating that the secondary metabolites had the activities of protease, amylase, and cellulase (Figure 4). The average diameter of the clear rings produced by protease activity was more than 0.82 cm, and that of amylase was more than 2.28 cm. Among all the strains tested, ZY133 had the strongest ability to produce protease and protease, generating rings of 1.90 cm and 2.69 cm in diameter, respectively, and that of cellulase was more than 1.98 cm. Z-14 had the strongest cellulase production capability.

### *Bacillus subtilis* strains Z-14 and JY214 are compatible

**Figure 5:**
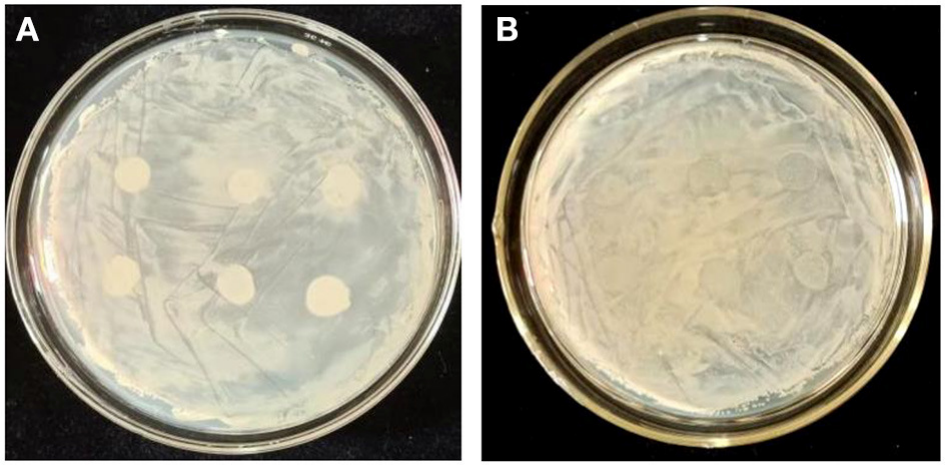
Determination of strain compatibility. **(A)** After coating Z-14 bacterial solution on NA medium, JY214 bacterial solution was added dropwise on top; **(B)** After coating JY214 bacterial solution, Z-14 bacterial solution was added dropwise on top.

Having identified that both Z-14 and JY214 have antagonistic effects against *Ggt*, we wondered if the two strains could function together to provide better performance. No transparent ring of mutual inhibition between strains was observed when Z-14 was coated on NA medium and JY214 was subsequently inoculated (Figure 5A), and there was no appearance of growth inhibition of the two tested bacteria when Z-14 was inoculated on the medium coated with JY214 (Figure 5B).

**Figure 6:**
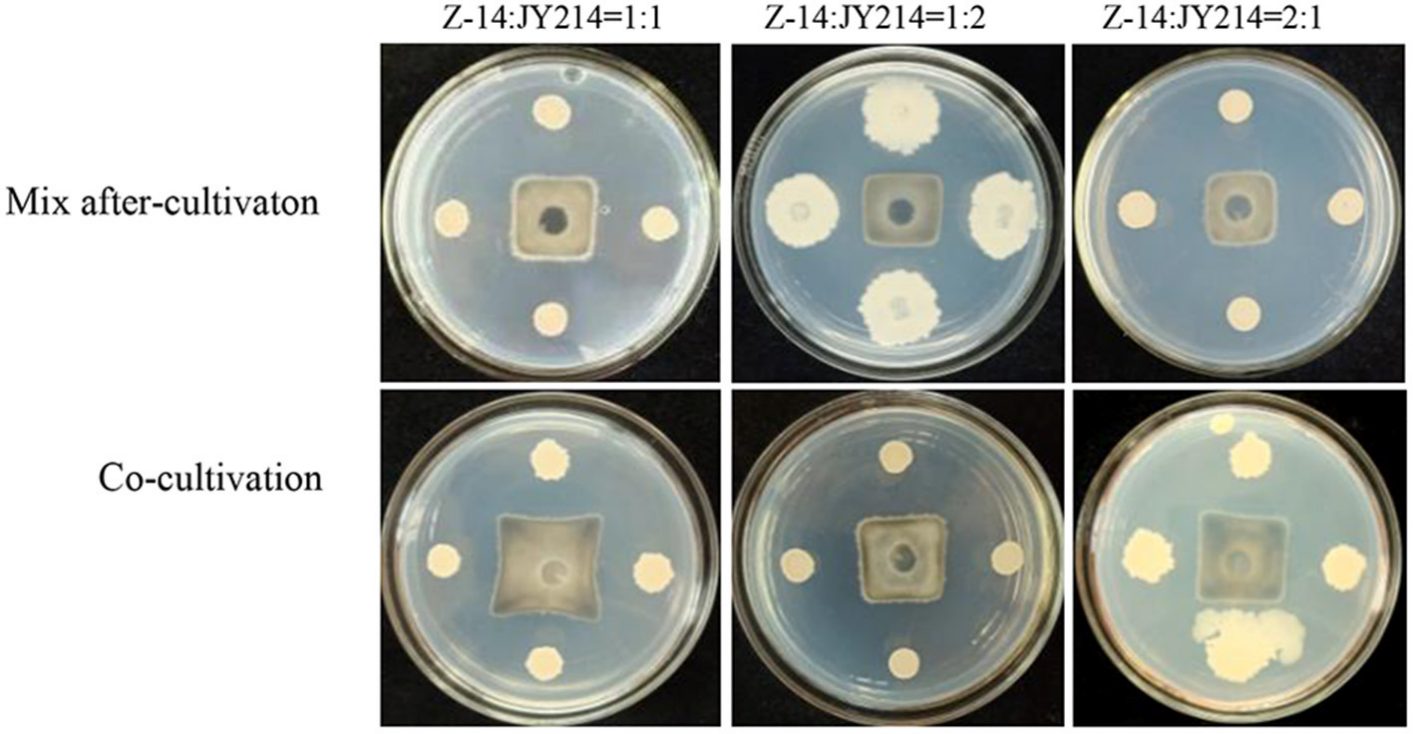
Inhibition test of different mixing method and ratios of Z-14 and JY214 against *Ggt*. Z-14 and JY214 were mixed in ratios of 1:1, 1:2, and 2:1 respectively to test the inhibition effect of the mixture on *Ggt*.

We subsequently tested the feasible mixing method and the optimal mixing ratio between Z-14 and JY214 (Figure 6). The mixing after-cultivation method of the two strains had a disease reduction rate of over 82% against *Ggt*, which was not only better than that produced by the co-cultivation method, but also superior to the effect of the two strains alone. Because Z-14 and JY214 could produce a reduction rate of 85.71% against *Ggt* when mixed at 2:1, we used the mixing ratio of 1:2 in the subsequent study (Table 2).

**Table 2:** Inhibition rates of different mixing method and ratios of Z-14 and JY214 against *Ggt*.

**Mixing method and ratio (Z-14:JY214)**		**Radius of the bacterial colony (cm)**	**Inhibition rate (%)**
Mix after-cultivation	1:1	0.74 ± 0.17a	82.34 ± 4.05b
	1:2	0.60 ± 0.03a	85.71 ± 0.60b
	2:1	0.73 ± 0.23a	82.74 ± 5.36b
Co-cultivation	1:1	1.13 ± 0.11b	73.02 ± 2.68a
	1:2	1.07 ± 0.01b	74.60 ± 0.34a
	2:1	1.06 ± 0.10b	74.80 ± 2.48a
	CK	-	-

The a and b different letters indicate the significant differences (*P* < 0.05 by one-way ANOVA and Duncan's multiple comparison).

### Effects of Z-14 and JY214 on mycelial growth of *Ggt*

**Figure 7:**
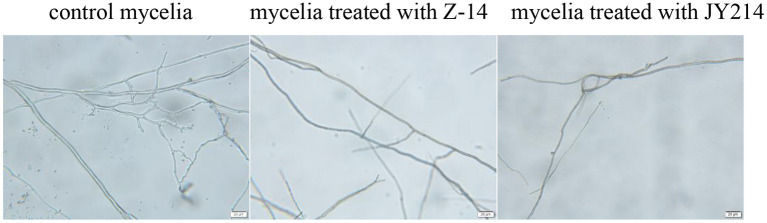
Inhibition of *Ggt* mycelial growth by Z-14 and JY214. The *Ggt* mycelia treated with Z-14 and JY214 were observed under a microscope. Bars = 20 μm.

We next investigated the effect of Z-14 and JY214 on the growth of *Ggt* mycelium. The mycelium of *Ggt* without inoculation with antagonistic *B. subtilis* had smooth surface, uniform morphology, normal development, with more branches, and the angle of branches was acute. After Z-14 treatment, the mycelium of *Ggt* were miscellaneous, unsaturated and generally thin, the branches were reduced and the angles of branches were uneven; the mycelium of *Ggt* treated with JY214 basically did not produce branches and the morphology of the main mycelium was changed, indicating that the growth of *Ggt* mycelium was inhibited under the influence of both Z-14 and JY214 (Figure 7).

### Determination of the control effect of antagonistic bacteria Z-14, JY214, and their combination on wheat take-all disease

**Figure 8:**
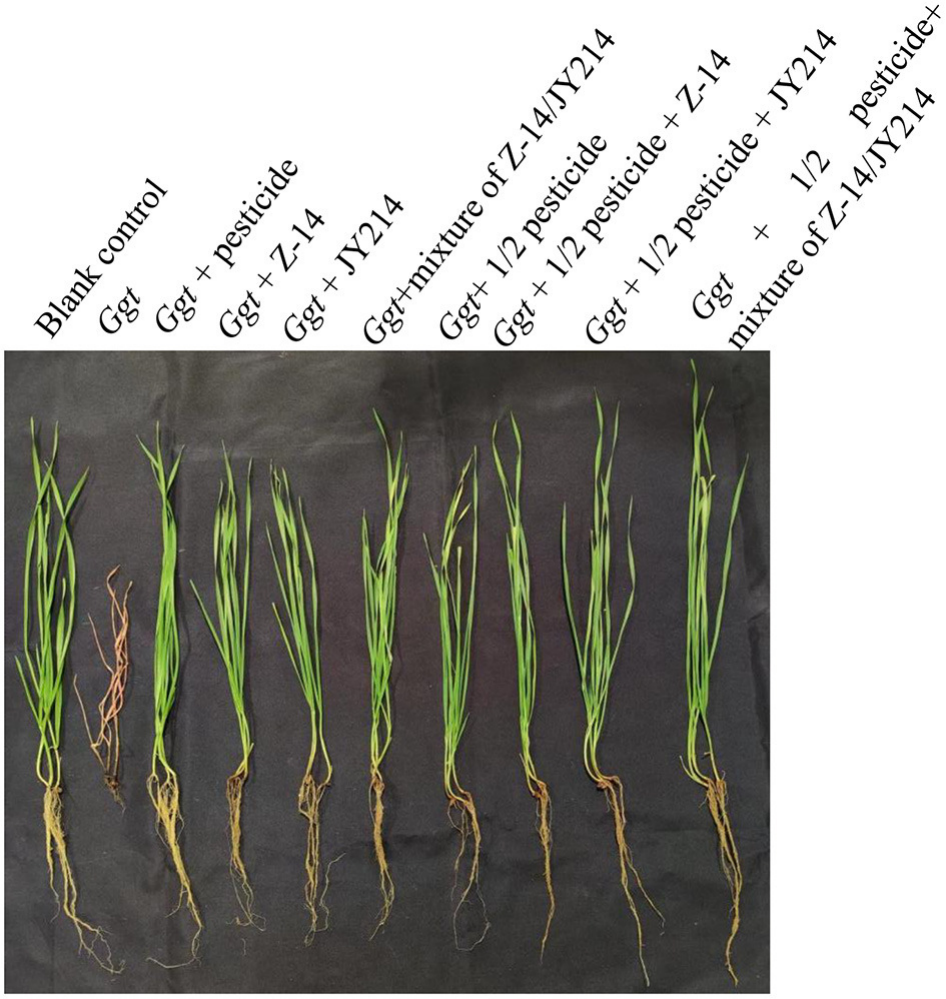
Control effect of pesticide, Z-14, JY214, and their combinations on wheat take-all disease. These treatments were conducted in the greenhouse.

To test the practical effects of Z-14 and JY214 on take-all diseases in wheat, we examined the effects of the two separately and combined in the greenhouse and in the field, and tested whether these combinations could reduce pesticide use. In the greenhouse experiments, the roots were washed 40 d after sowing and the disease condition was checked. In the control group that was inoculated with *Ggt* only, all the wheat seedlings withered, the disease index of take-all disease reached 100%, while the seedlings treated with pesticide had lower disease-index, and the disease-index of all treatmental groups was reduced compared to the control group inoculated with *Ggt* only (Table 3). Application of Z-14 or JY214 reduced the disease index induced by *Ggt* infection to 34–40.63%, indicating that application of both Z-14 and JY214 exerted control on take-all disease, yielding a 59.37–65.62% disease reduction rate. Combined application of Z-14 and JY214 suppresses take-all disease better than their individual effects (Figure 8). As compared to the group with only reducing the pesticide application by 50%, the disease reduction rate reached 68.75%, 69.79%, and 73.96%, by adding Z-14 application, JY214 application, or the co-application of Z-14 and JY214, respectively. Therefore, the application of *B. subtilis* with a reduced dosage of pesticide by half can better control take-all disease. We further measured the effects of Z-14 and JY214 on three biomass characters, wheat plant height, stem width, and root length. Since all wheat seedlings treated with *Ggt* were withered, we recorded all their stem widths as 0 mm. Application of either Z-14 or JY214 (or a mixture of the two strains) on the basis of a 50% reduction in pesticide application reduced wheat growth by *Ggt* (Table 4), with the same results as in our assessment of disease reduction rate in a greenhouse environment.

**Table 3:** Control effect of pesticide, Z-14, JY214, and their combinations on wheat take-all disease in the greenhouse experiments.

**Treatments**	**Disease index (%)**	**Reduction rate (%)**
Blank control	0	-
*Ggt*	100	-
*Ggt +* pesticide	30.21	69.79
*Ggt* + Z-14	40.63	59.37
*Ggt* + JY214	39.58	60.42
*Ggt* + mixture of Z-14/JY214	34.38	65.62
*Ggt* + 1/2 pesticide	36.46	63.54
*Ggt* + 1/2 pesticide + Z-14	31.25	68.75
*Ggt* + 1/2 pesticide + JY214	30.21	69.79
*Ggt* + 1/2 pesticide + mixture of Z-14/JY214	26.04	73.96

**Table 4:** Effects of pesticide, Z-14, JY214, and their combinations on root length, root fresh weight, and aboveground weight of *Ggt*-infected wheat in the greenhouse experiments.

**Treatments**	**Stem width (mm)**	**Plant height (cm)**	**Root length (cm)**
Blank control	1.40 ± 0.04e	30.31 ± 0.76d	14.54 ± 0.40c
*Ggt*	0.00 ± 0.00a	6.23 ± 0.57a	1.94 ± 0.13a
*Ggt +* pesticide	1.31 ± 0.01b	28.46 ± 0.58c	13.42 ± 0.42bc
*Ggt* + Z-14	1.34 ± 0.01c	26.88 ± 0.33b	13.52 ± 0.75bc
*Ggt* + JY214	1.37 ± 0.02cd	26.54 ± 0.61b	13.83 ± 0.18bc
*Ggt* + mixture of Z-14/JY214	1.35 ± 0.02cd	26.52 ± 0.13b	13.04 ± 0.58b
*Ggt* + 1/2 pesticide	1.35 ± 0.02cd	28.44 ± 0.56c	13.83 ± 0.94bc
*Ggt* + 1/2 pesticide + Z-14	1.36 ± 0.02cd	31.13 ± 0.22d	13.25 ± 0.70b
*Ggt* + 1/2 pesticide + JY214	1.37 ± 0.01cde	32.06 ± 0.38e	13.75 ± 0.45bc
*Ggt* + 1/2 pesticide + mixture of Z-14/JY214	1.39 ± 0.00de	32.44 ± 0.82e	13.67 ± 0.83bc

The a–e different letters indicate the significant differences (*P* < 0.05 by one-way ANOVA and Duncan's multiple comparison).

To imitate a wheat production environment, we conducted experiments in the field to examine the effect of Z-14 and JY214 on the disease-index of *Ggt*-induced take-all disease and its disease-reduction rate, and measured the biomass characters (Table 5). After inoculation with *Ggt*, the disease index of take-all in wheat reached 34%. Application of Z-14 on the basis of a 50% reduced dosage of pesticide resulted in the lowest disease-index with a disease reduction rate of 72.12%, while application of a mixture of the two strains, Z-14 and JY214, on the basis of a 50% pesticide application resulted in a take-all disease-index of 16.96% and the disease-reduction rate was 50.12%. In the absence of pesticide application, Z-14, JY214, or their combination could also reduce the disease level of wheat effectively with a disease reduction rate between 51.82 and 54.76%, which was even slightly higher than the disease reduction rate when only pesticide was applied.

**Table 5:** Control effect of pesticide, Z-14, JY214, and their combinations on wheat take-all disease in the field experiments.

**Treatments**	**Disease index (%)**	**Reduction rate (%)**
Blank control	0	-
*Ggt*	34.00	-
*Ggt +* pesticide	16.67	50.97
*Ggt* + Z-14	16.00	52.94
*Ggt* + JY214	16.38	51.82
*Ggt* + mixture of Z-14/JY214	15.38	54.76
*Ggt* + 1/2 pesticide + Z-14	9.48	72.12
*Ggt* + 1/2 pesticide + mixture of Z-14/JY214	16.96	50.12

Furthermore, field applications of both the strains of *B. subtilis* in wheat helped to restore the biomass decline caused by *Ggt*. All the other groups tested showed higher plant height, root length, and stem width compared to the experiment in which only *Ggt* was inoculated (Table 6). We next measured wheat yields in each treatment and the yield of wheat after *Ggt* inoculation was 7,176.05 ton/ha, representing a 10.60% yield reduction relative to the untreated wheat. All the treatments in which *B. subtilis* or pesticides were applied restored the yield of *Ggt*-affected wheat to a certain extent, with a high yield of 8,475.60 ton/ha in the treatment in which Z-14 was applied on the basis of 50% pesticide application. The use of *B. subtilis* and halving treatment with pesticides not only provides better control of wheat total erosion disease, but also maintains and even increases wheat yield (Table 7).

**Table 6:** Effects of pesticide, Z-14, JY214, and their combinations on root length, root fresh weight, and aboveground weight of *Ggt*-infected wheat in the field experiments.

**Treatments**	**Stem width (cm)**	**Plant height (cm)**	**Root length (cm)**
Blank control	0.40 ± 0.01cd	74.43 ± 1.49c	14.97 ± 1.82c
*Ggt*	0.34 ± 0.02a	65.13 ± 3.26a	11.26 ± 0.49a
*Ggt +* pesticide	0.39 ± 0.01bcd	69.50 ± 1.25b	14.06 ± 1.04bc
*Ggt* + Z-14	0.41 ± 0.02d	68.10 ± 1.16b	12.75 ± 0.65ab
*Ggt* + JY214	0.37 ± 0.02b	65.10 ± 0.97a	12.29 ± 1.72ab
*Ggt* + mixture of Z-14/JY214	0.38 ± 0.02bc	63.33 ± 2.21a	11.15 ± 1.46a
*Ggt* + 1/2 pesticide + Z-14	0.37 ± 0.02b	65.25 ± 1.10a	13.18 ± 1.10abc
*Ggt* + 1/2 pesticide + mixture of Z-14/JY214	0.40 ± 0.02cd	68.92 ± 1.00b	12.86 ± 1.36ab

The a–d different letters indicate the significant differences (*P* < 0.05 by one-way ANOVA and Duncan's multiple comparison).

**Table 7:** Effects of pesticide, Z-14, JY214, and their combinations on yield of *Ggt*-infected wheat in the field treatments.

**Treatments**	**Number of ears per m^2^**	**Number of grains per ear**	**1,000-grain weight /g**	Yield^*^/ton·ha^−1^
Blank control	521.00	41.67	43.50	8,027.30
*Ggt*	485.00	40.90	42.56	7,176.05
*Ggt +* pesticide	503.00	42.33	43.50	7,872.71
*Ggt* + Z-14	509.00	42.33	43.10	7,893.37
*Ggt* + JY214	513.00	42.00	42.80	7,838.43
*Ggt* + mixture of Z-14/JY214	511.00	42.40	43.20	7,955.90
*Ggt* + 1/2 pesticide + Z-14	517.00	42.67	45.20	8,475.60
*Ggt* + 1/2 pesticide + mixture of Z-14/JY214	509.00	42.33	43.00	7,875.05

^*^Yield = Number of ears per ha × number of grains per ear × 1,000-grain weight (kg) × 85% / 10^6^.

## Discussion

Biocontrol by bacteria treatment has the advantages of eco-friendly, high efficiency, safety and sustainability, which has become a research hotspot of plant disease control and has broad application prospects (Zhang et al., 2022). *Bacillus* spp. are extensively found in the environment and have been found to produce a multitude of secondary metabolites, some of which (including proteases, cellulases, amylases, etc.) act as fungicides to inhibit the growth of pathogenic bacteria (Sun et al., 2003; Romero et al., 2007; Brzezinska et al., 2020).

Wang et al. (2020) found that *B. amyloliquefaciens* HG01 attained control of anthracnose rot in tomato by inhibiting the growth of *Colletotrichum acutatum*. Tanaka et al. (2017) detected significant inhibition of cucumber powdery mildew by *B. amyloliquefaciens* SD-32. Chien and Huang (2020) showed that foliar sprays of *B. amyloliquefaciens* and *Trichoderma asperellum* significantly enhanced the biocontrol of bacterial spot disease in tomato. Suspension of *Bacillus* spp. strain DR-08, SC, was effective in controlling tomato brucellosis (Seong et al., 2020). Zheng et al. (2018) found that the inhibitory protein produced by *B. subtilis* Zl-2 could deform the mycelial morphology of *Fusarium graminearum* and inhibit its spore germination. In this study, all the five bacterial strains tested produce protease, cellulase and amylase, indicating that the strains could degrade fungal cell walls, destroy fungal cells and inhibit the growth of pathogenic mycelia so as to achieve the purpose of controlling pathogenetic infection. It was observed that strains Z-14 and JY214 changed the morphology of mycelia of *Ggt*, but whether they affected the internal organelles required to be further examined by transmission electron microscopy.

At present, the reports on biological control of diseases focus mainly on single biocontrol strain. Predictably, however, preparations made from single strains have solely antibacterial mechanisms and are susceptible to a variety of biotic and abiotic factors, which makes it difficult to give full play to its antibacterial effect and potential biocontrol function. Compound biocontrol agents can achieve the advantages of complementation among multiple strains, enhance the natural competitiveness and viability, improve the effect and stability of disease control, and inhibit better the growth of pathogenic bacteria through the synergistic effect of a variety of antibacterial mechanisms (Xu et al., 2010). The combination of *B. subtilis* EPCO16 and EPC5 with *Pseudomonas fluorescens* Pf1 was more effective in controlling disease caused by *Fusarium solani* than by themselves alone (Sundaramoorthy et al., 2012). Yu et al. (2015) compounded *B. subtilis* B41 and *Pseudomonas putida* B57 to control black shank disease in tobacco and achieved 75.72% control efficiency in greenhouse experiment and 67.99% control efficiency in the field experiment. The control efficiency was higher than that produced by using single strains. In the present study, application of Z-14 and JY214 alone and a combination of the two was effective in controlling take-all disease. The individual and combined use of Z-14 and JY214 did not produce differences in disease control effects, probably because of the high level of inhibition of *Ggt* produced by Z-14 and JY214 alone, and the advantage of the combination of the two strains was not uncovered under such experimental conditions.

Certain *Bacillus* spp. are capable of inducing resistance in plants and improving their resistance to diseases and stresses (Zhang et al., 2014b; Tang et al., 2016). Although not numerous, the use of bacterial strains with biocontrol effects in combination with pesticides (or resistance inducers) have been reported. The combination of *Trichoderma* with resistance inducers provided control of cotton root rot (Abo-Elyousr et al., 2009), and the combination of four strains of plant growth-promoting inter-rhizobacteria and hymexazol was more effective in plant growth promotion and control of Fusarium crown and root rot in tomato (Myresiotis et al., 2012). Our findings that the combined application of *B. subtilis* strains and pesticides can be more effective in controlling plant diseases are consistent with these reports.

Commercialization and large-scale application of some microorganisms with biocontrol effects are being achieved. For example, *Bacillus* strain QST-713 (Serenade^TM^) promotes the growth of tomato plants and protects them from salt stress and *Fusarium oxysporum* f. sp. *lycopersici* attack (Medeiros and Bettiol, 2021). In greenhouse and field experiments, the application of the commercial biocontrol treatment Stargus (with the active ingredient *B. amyloliquefaciens* F727) was effective in controlling the severity of crown and root rot induced by *Phytopythium vexans* infesting ginkgo and red maple plants (Panth et al., 2021). It is foreseeable that the strains demonstrated in this study and their combinations with pesticides are promising for certain applications.

## Conclusion

In this study, it was revealed that two *B. subtilis* strains, Z-14 and JY214, function to inhibit the growth of *Ggt*. Z-14 and JY214 not only produce secretions with protease, amylase and cellulase activities, but also promote wheat growth. The combination of Z-14 and JY214 allows for the control of wheat take-all disease in the field with a reduction in the use of the pesticide Koras. The reported solution mitigates the risk of potential environmental damage from the pesticide and reduces the adverse effect of the disease on wheat yield.

## Data availability statement

The original contributions presented in the study are included in the article/supplementary material, further inquiries can be directed to the corresponding authors.

## Author contributions

DW and SH supervised the project. CH and DZ conceived and designed the experiments. GZ, ZZ, JZ, and YB performed the experiments and analyzed the data. GZ and TS wrote the manuscript. All authors contributed to the article and approved the submitted version.
